# Age Differences in Engagement in Hong Kong Anti-Extradition Bill Protest: The Role of Self-Relevance

**DOI:** 10.1093/geroni/igaa057.1090

**Published:** 2020-12-16

**Authors:** Tze Kiu Wong, Dwight C K Tse, Nicole Long Ki Fung, Helene Fung

**Affiliations:** 1 The Chinese University of Hong Kong, Hong Kong, China; 2 the Chinese University of Hong Kong, Hong Kong, China; 3 The Chinese University of Hong Kong, Sha Tin, China

## Abstract

Older adults were found to be less involved in non-institutional political actions than younger people did, and our previous work found that self-relevance mediated this age difference. In this study, we attempted to replicate the finding in a real-life social movement. We recruited 1037 participants (aged 18-84) during the anti-extradition bill movement in Hong Kong in September 2019. They responded to questions of how relevant and important the movement was to them, and whether they had taken part in a list of 8 political actions (e.g. signing petitions, joining rallies). Older adults indeed participated less in the movement compared with younger adults, and the age difference could partly be attributed to a lower perceived relevance of the movement. The finding suggested emphasizing on self-relevance as a potential way to promote political participation in older adults.

